# Chromosomal painting in *Charadrius collaris* Vieillot, 1818 and *Vanellus chilensis* Molina, 1782 and an analysis of chromosomal signatures in Charadriiformes

**DOI:** 10.1371/journal.pone.0272836

**Published:** 2022-08-10

**Authors:** Melquizedec Luiz Silva Pinheiro, Cleusa Yoshiko Nagamachi, Talita Fernanda Augusto Ribas, Cristovam Guerreiro Diniz, Patricia Caroline Mary O´Brien, Malcolm Andrew Ferguson-Smith, Fengtang Yang, Julio Cesar Pieczarka

**Affiliations:** 1 Laboratório de Citogenética, Centro de Estudos Avançados da Biodiversidade, ICB, Universidade Federal do Pará, Belém, Pará, Brazil; 2 Laboratório de Biologia Molecular e Neuroecologia, Instituto Federal do Pará, Campus de Bragança, Bragança, Pará, Brazil; 3 Department of Veterinary Medicine, Cambridge Resource Centre for Comparative Genomics, University of Cambridge, Cambridge, United Kingdom; 4 Cytogenetics Facility, Wellcome Trust Sanger Institute, Hinxton, Cambridgeshire, United Kingdom; Universidade Federal de Ouro Preto, BRAZIL

## Abstract

Charadriiformes represent one of the largest orders of birds; members of this order are diverse in morphology, behavior and reproduction, making them an excellent model for studying evolution. It is accepted that the avian putative ancestral karyotype, with 2n = 80, remains conserved for about 100 million years. So far, only a few species of Charadriiformes have been studied using molecular cytogenetics. Here, we performed chromosome painting on metphase chromosomes of two species of Charadriidae, *Charadrius collaris* and *Vanellus chilensis*, with whole chromosome paint probes from *Burhinus oedicnemus*. *Charadrius collaris* has a diploid number of 76, with both sex chromosomes being submetacentric. In *V*. *chilensi* a diploid number of 78 was identified, and the Z chromosome is submetacentric. Chromosome painting suggests that chromosome conservation is a characteristic common to the family Charadriidae. The results allowed a comparative analysis between the three suborders of Charadriiformes and the order Gruiformes using chromosome rearrangements to understand phylogenetic relationships between species and karyotypic evolution. However, the comparative analysis between the Charadriiformes suborders so far has not revealed any shared rearrangements, indicating that each suborder follows an independent evolutionary path, as previously proposed. Likewise, although the orders Charadriiformes and Gruiformes are placed on sister branches, they do not share any signature chromosomal rearrangements.

## Introduction

Charadriiformes represent one of the largest orders of birds, with 19 families and 383 species. This order is divided into three large monophyletic suborders: Charadrii (plovers), Scolopaci (snipes, partridges) and Lari (seagulls, terns and mandrels) [1]. Members of this order are quite diverse in morphology, behavior and reproduction, which make them an excellent model for studying evolution in different groups [2].

Molecular data analyses suggest that these three suborders arose in the late Cretaceous and that at least 14 modern Charadriiformes lineages have survived the late Cretaceous mass extinction [3]. Molecular data reveal that Lari and Scolopaci are sister branches and Charadrii is in a more basal position [1].

It is accepted that the avian putative ancestral karyotype (PAK) with 2n = 80, remained conserved for about 100 million years, with few variations in Neoaves [4, 5]. Although the order Charadriiformes has 383 species, only 64 species (16.7%) from eleven families have had their karyotypes studied by classical cytogenetics [6–9]. This order has a huge karyotype diversity, with the family Charadriidae having a diploid number ranging from 58 to 78 [10, 11]. This makes it of interest to study its evolutionary process from a cytogenetics point of view. Chromosome painting has been used in identifying such chromosomal diversity [12]. In particular, whole chromosome probes made from the species *Burhinus oedicnemus* (Burhinidae, Charadriiformes), in which most microchromosomes are fused, therefore provide improved resolution for comparative analysis in Charadriiformes [13]. Chromosome painting studies between BOE and GGA shows the conservation of pairs GGA1, GGA2, GGA3, GGA4q and GGA5, but with the presence of some intrachromosomal rearrangements, probably pericentric inversions [13]. BOE7 to BOE14 show microchromosome homologies. Pericentric inversion or centromere repositioning lead to the morphological change in some pairs of autosomes [7, 11, 14] and in the sex chromosomes Z and W [15]. However, only five studies in Charadriiformes using molecular cytogenetics have been carried out so far [13, 16–19].

As Charadriiformes is an order where the phylogenetic relationships of a major higher-level clade have been successfully resolved [1], comparative chromosome painting analysis of the three suborders using BOE painting probes could shed new light on phylogenetic relationships and define karyotype evolution in the order. Our hypothesis is that this approach will allow us to find chromosome signatures specific for each suborder. Since the order Gruiformes is a sister branch of Charadriiformes [1] and that BOE and GGA chromosomal painting data exist for Gruiformes [16, 20, 21, 28], we expect that this phylogenetic proposition can be confirmed.

The aim of the present paper is to undertake comparative analysis between the three suborders of Charadriiformes and the order Gruiformes by homology mapping of the two species of Charadriidae, *Vanellus chilensis* (VCH) and *Charadrius collaris* (CCO). The results are compared with those obtained in *Gallus gallus* [22], *Burhinus oedicnemus* [13], *Larus argentatus* Pontoppidan 1763 [16], *Actitis macularius* Linnaeus, 1766 [18] and *Jacana jacana* Linnaeus, 1766 [17]. We also searched for a chromosomal signature that would connect the orders Charadriiformes and Gruiformes.

## Material and methods

### Ethics statement

The specimens were kept stress-free with full access to food and water until euthanasia was performed in accordance with animal welfare guidelines established by Brazilian resolution CFMV n.1000/2012. The necessary euthanasia was performed by intraperitoneal injection of buffered and diluted barbiturates after local anesthesia, in accordance with animal welfare guidelines established by the Animal Ethics Committee (Comitê de Ética Animal) from Universidade Federal do Pará (UFPA), which authorized the present study (Permit 68–2015). JCP has a permanent field permit, number 13248 from “Instituto Chico Mendes de Conservação da Biodiversidade”. The Cytogenetics Laboratory from UFPA has a special permit number 19/2003 from the Ministry of Environment for samples transport and 52/2003 for using the samples for research.

### Sampling

#### Charadrius collaris

**Fig 1:**
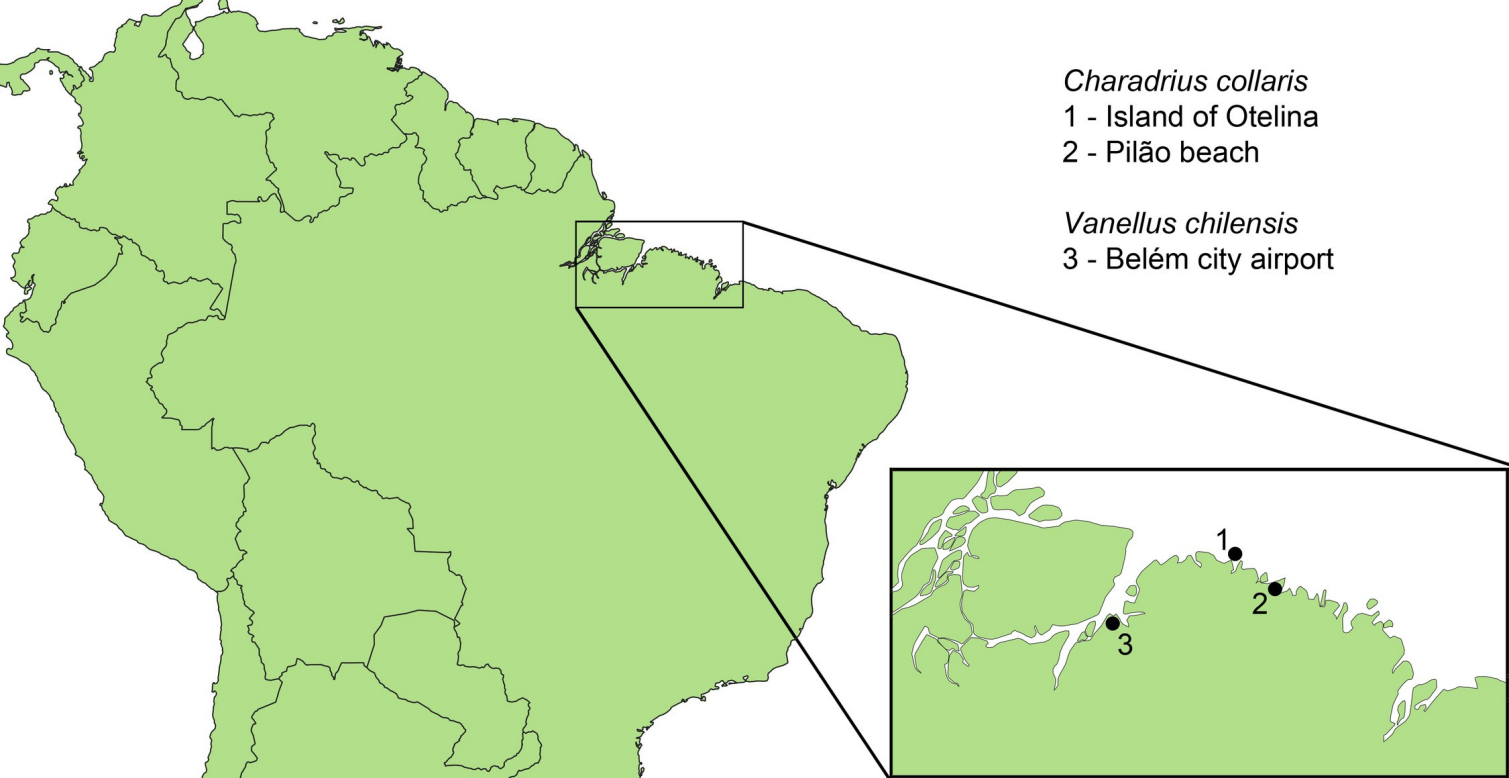
Map of geographical distribution of *Charadrius collaris* and *Vanellus chilensis*, with their respective collection locations. *Charadrius collaris*: 1-Island of Otelina; 2- Pilão beach; *Vanellus chilensis*: 3-Belém Airport. The map was prepared using the QUANTUM-GIS software, v. 2.10.1 (https://qgis.org). The free database was obtained from DIVA-GIS (https://www.diva-gis.org).

Samplings were carried out in conjunction with the Laboratory of Molecular and Environmental Biology of the Federal Institute of Pará (IFPA)—Campus Bragança, which provided all technical support. They were performed in Otelina island (0°45’42.57"S; 46°55’51.86"W, one male and one female) and Pilão beach (0°47’46.08"S; 46°40’29.64"W, two females), on the coast of the Northeast Region of Pará, Brazil (Fig 1). Nets with 12m x 2m and 36mm mesh were used at six collection points.

#### Vanellus chilensis

The samples were collected at the international airport Val—de—Cans in Belém (SBBE) which covers an area of 631 ha, located 12 km from the center of Belém city, with coordinates 01°23´04´´S and 48°28´42´´W (Fig 1). Four eggs were collected for the cultivation of embryonic cells. Karyotypic analysis showed that they were all males.

### Cytogenetic analysis

Metaphase chromosomes were obtained by the bone marrow technique [23] for CCO. Briefly, 0.05% aqueous colchicine was first injected intraperitoneally at the dosage of 0.01 ml per 10 g body weight. The animal was sacrificed by overdose of lidocaine (20mg/ml) injected intraperitoneally. The femur was extracted and the bone marrow was removed. The bone marrow was placed in a homogenizer and incubated in a hypotonic solution (KCl 0.075 M) for 20–30 minutes at 37°C. Then, 1 mL of ice-cold Carnoy fixative (methanol and glacial acetic acid in a 3:1 ratio) was added.

The chromosomes of VCH were obtained from embryonic cell cultures [24]. Eggs were collected and placed in an incubator at 38°C. After 40 hours the eggs blastodisks were transferred to culture medium with addition of colcemide, followed by a 3 hours culture. The following (hypotonic solution, fixation and slide preparation) are the same as the bone marrow technique.

The chromosomes were classified in decreasing size according to the suggested nomenclature [25]. Standard G-banding was performed [26].

### Chromosome painting

Whole chromosome probes from BOE generated by flow cytometry [13] were used in this study. Fluorescence *in situ* hybridization was performed according to Yang et al. [27]. Single and double hybridization experiments were carried out, combining probes labeled with biotin and detected with Avidin conjugated with Cy3 or probes labeled with digoxigenin and detected with anti-digoxigenin conjugated with fluorescein. Counter staining was with DAPI (4’6-Diamidino-2-phenyl-indole). Slides were analyzed in a Nikon H550S microscope, with a DS-Qi1Mc digital camera controlled by the Nis-Elements software. The images were captured in black and white and subsequently pseudo-colored based on the fluorochrome used. Images were edited with the Adobe Photoshop CS6 software.

## Results

### Karyotype description and chromosomal painting in *Charadrius collaris*

**Fig 2:**
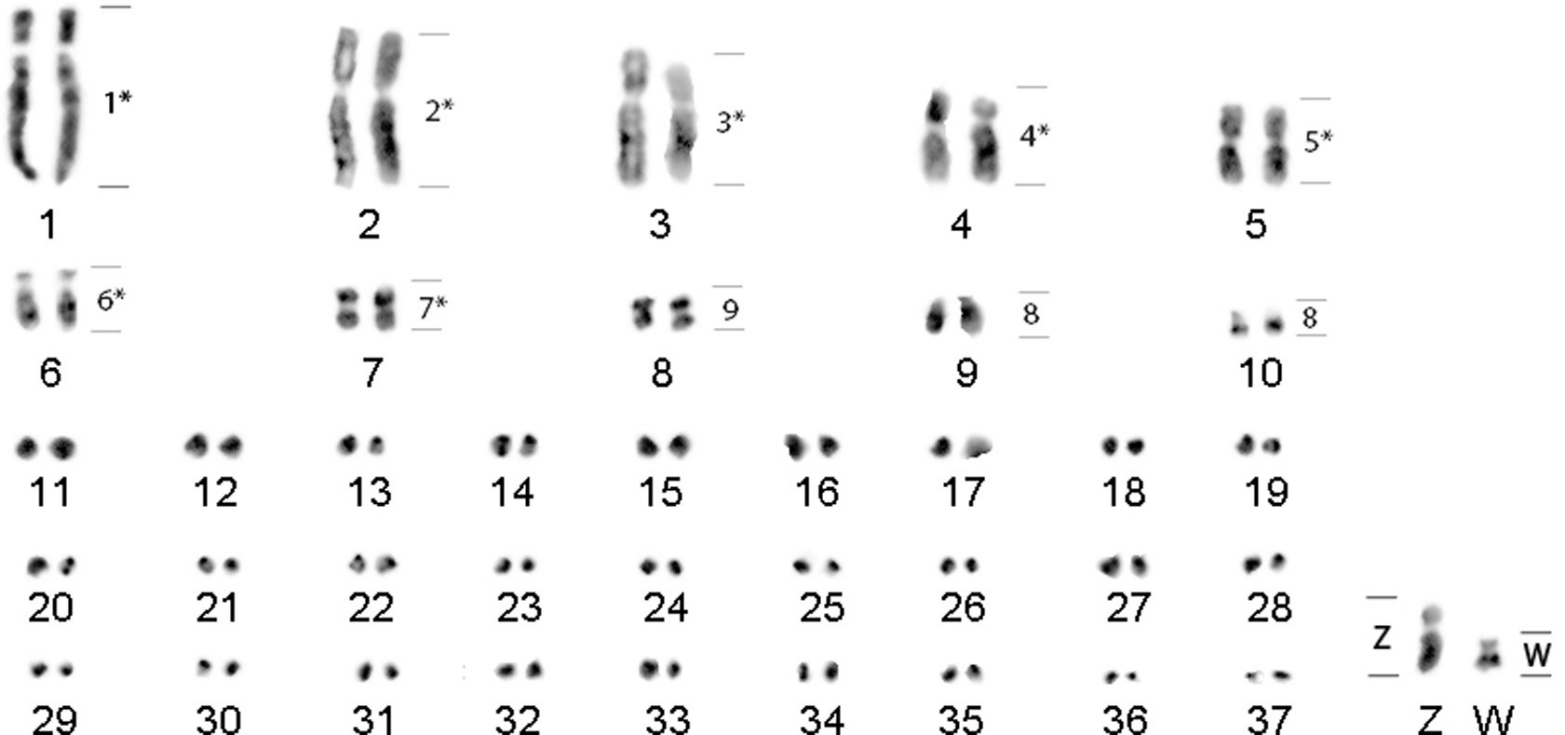
G-banding in *Charadrius collaris* (2n = 76) showing the homologies of whole chromosome probes of *Burhinus oedicnemus* (right). (*) Macrochromosome probes reveal homeology to the sex chromosome W. The microchromosomes are arranged sequentially, as the correct homeologies cannot be detected due to the lack of reliable markers.

*Charadrius collaris* has a diploid number (2n) of 76, where pairs 5, 7 and 8 are metacentric; pairs 1–4 are submetacentric; pair 6 is acrocentric and the remaining pairs are telocentric; the others are microchromosomes (Fig 2). Sex chromosomes are ZZ/ZW. Females have a sub-metacentric Z chromosome, intermediate in size between autosomal pairs 5 and 6. The W chromosome is a small sub-metacentric, similar in size to autosomal pair 9. Hybridizations with BOE whole chromosome probes confirmed that these are the sex chromosomes (Fig 2).

**Fig 3:**
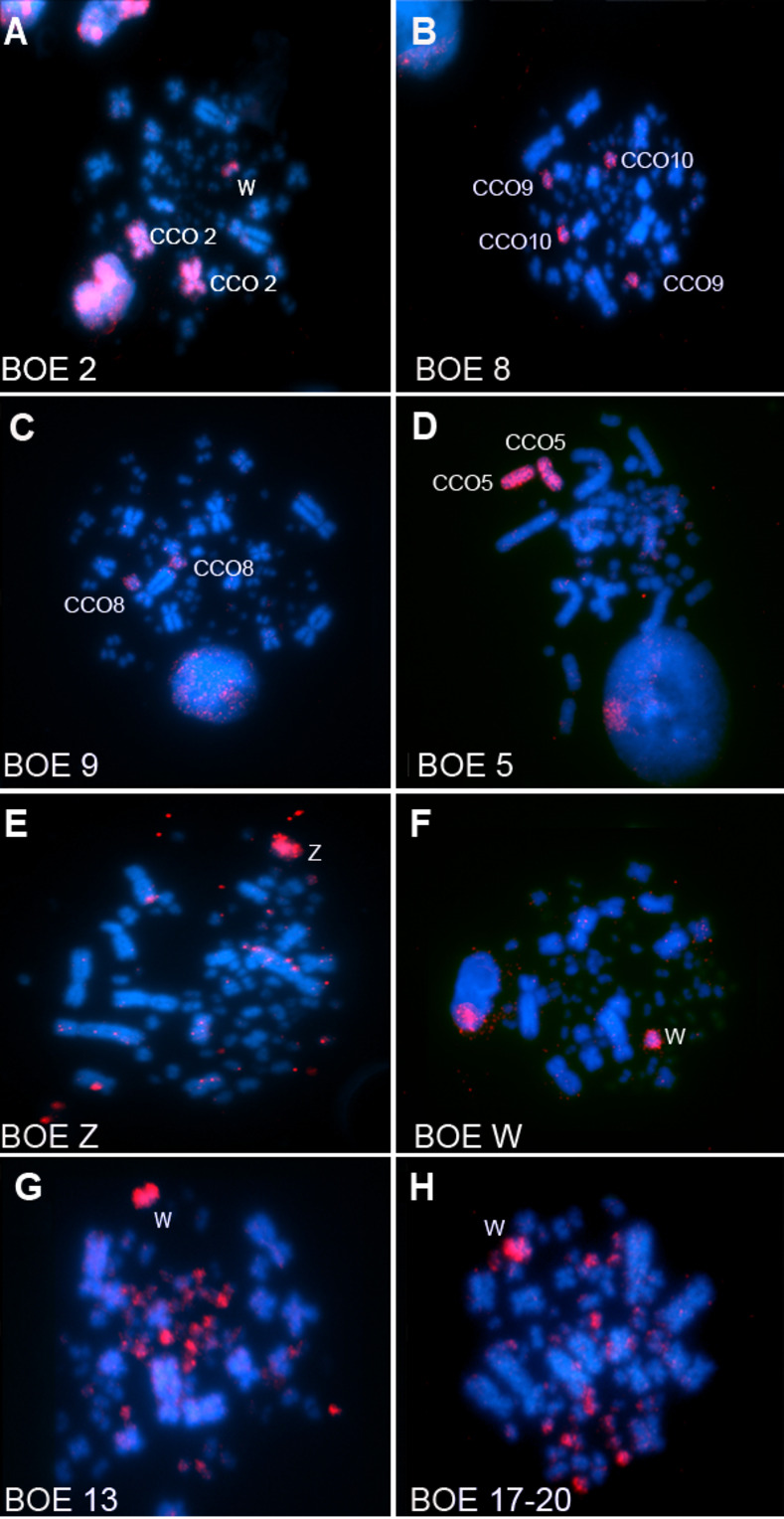
Chromosomal painting of whole chromosome probes of *Burhinus oedicnemus* on *Charadrius collaris*. The probes are visualized with avidin-Cy3 (red); chromosomes counterstained with DAPI (blue).

The FISH experiments with BOE whole chromosome probes in CCO revealed the following homologies: each one of the pairs CCO1 (BOE1), CCO2 (BOE2), CCO3 (BOE3), CCO4 (BOE4), CCO5 (BOE5), CCO6 (BOE6), CCO7 (BOE7) and CCO8 (BOE9) were equivalent to a pair of chromosomes per BOE probe. Each of these probes also showed hybridization to the sex chromosome W. The BOE8 probe showed homology to CCO9 and CCO10. The sex chromosome probes hybridized only to the sex chromosomes of CCO. Seven probes hybridized to microchromosomes: BOE10 (2 micros), BOE11 (4 micros), BOE12 (4 micros), BOE13 (20 micros), BOE14 (2 micros) and BOE17-20 (12 micros) and, with the exception of BOE10 and BOE11 they also hybridized to the W chromosome. Examples of hybridizations are shown in Fig 3.

### Karyotype description and chromosomal painting in *Vanellus chilensis*

**Fig 4:**
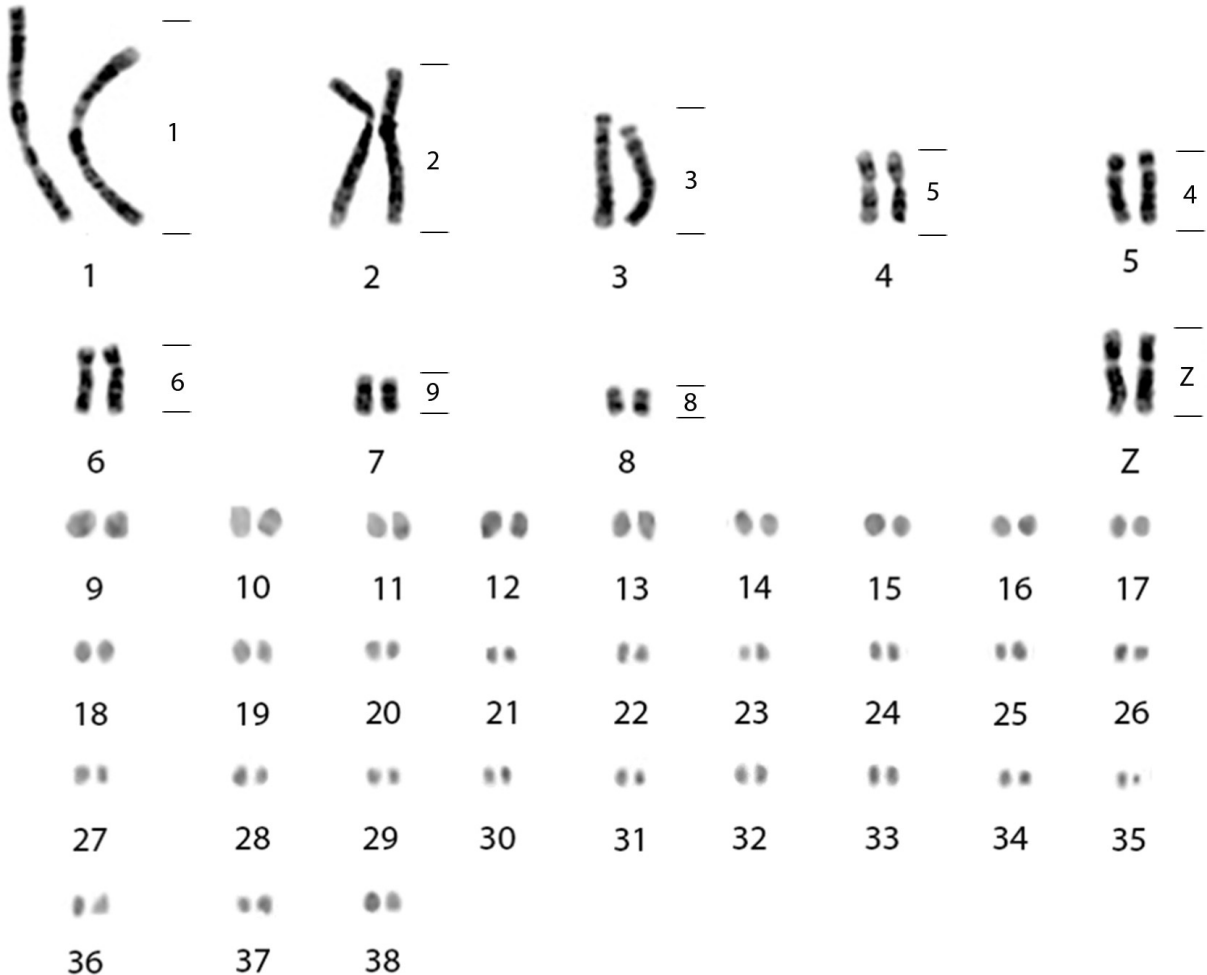
G-banding in *Vanellus chilensis* (2n = 78) showing the homologies of whole chromosome probes of *Burhinus oedicnemus* (right). The microchromosomes are arranged sequentially as the correct homeologies cannot be detected due to lack of reliable markers.

*Vanellus chilensis* has a diploid number of 78, where the first, fourth, seventh and eighth pairs are metacentric; the second, fifth and sixth pairs are submetacentric; the third pair is subtelocentric and the remaining pairs are microchromosomes. The sex chromosome system is of the ZZ/ZW type. VCH samples were obtained from eggs. All karyotypes were ZZ, indicating that the birds were male. The Z chromosome is submetacentric, almost metacentric, and is the same size as the 4th pair (Fig 4). Hybridizations with BOE whole chromosome probes confirmed that this chromosome is the Z (Fig 4).

**Fig 5:**
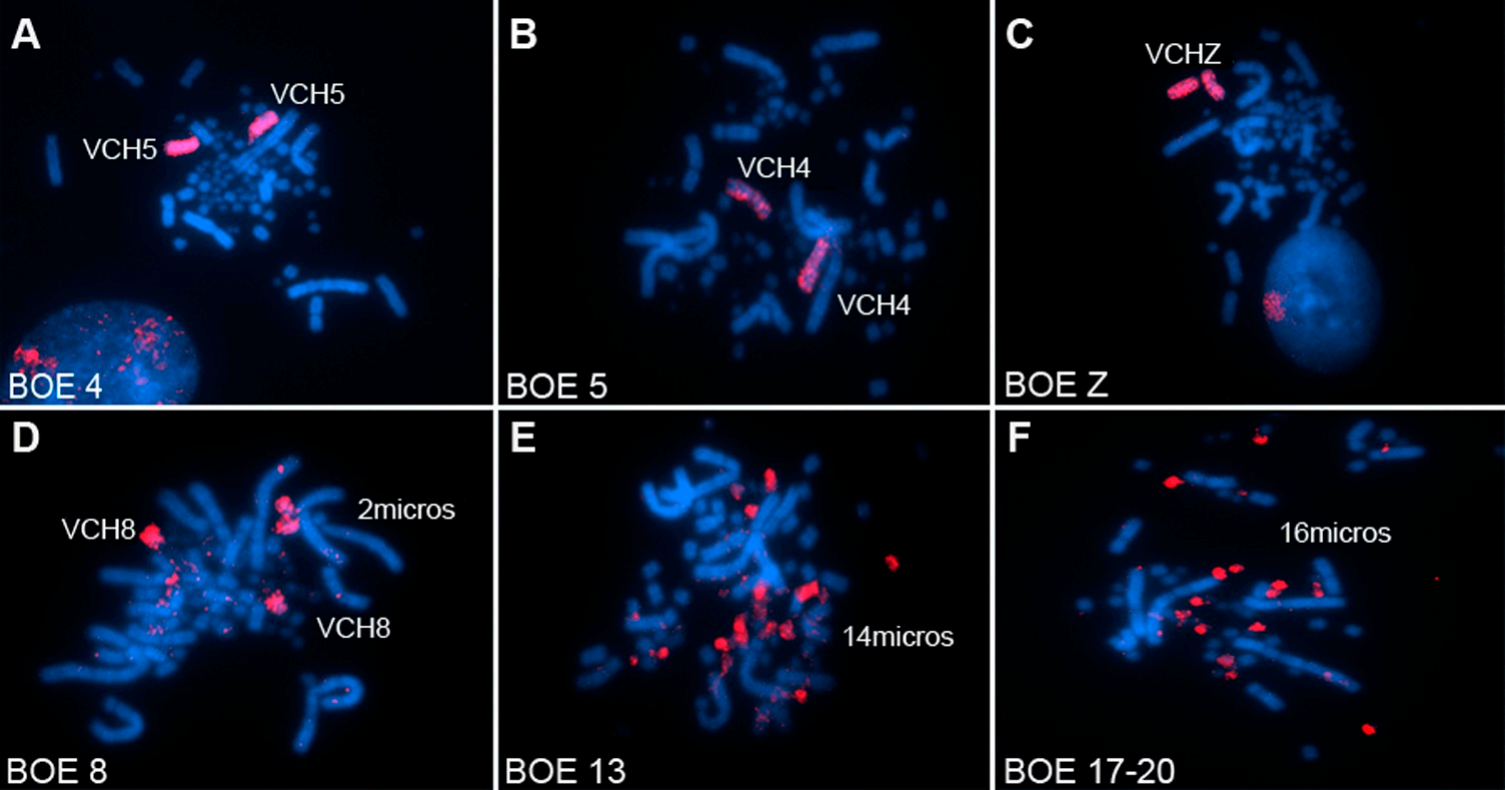
Chromosomal painting of whole chromosome probes of *Burhinus oedicnemus* homologous to macrochromosomes in *Vanellus chilensis*. The probes are visualized with avidin-Cy3 (red); chromosomes counter-stained with DAPI (blue).

The FISH experiments with BOE whole chromosome probes in VCH demonstrated homology of BOE1, BOE2, BOE3, BOE5, BOE4, BOE6 and BOE9 with VCH1, VCH 2, VCH3, VCH4, VCH5, VCH6 and VCH7 respectively; BOEZ probe hybridized to VCHZ. The BOE7- 8, BOE10-14 and BOE17-20 showed some homology to microchromosomes. BOE7 hybridized to 7 microchromosomes; BOE8 hybridized to VCH8 + 2 microchromosomes. BOE10-11 hybridized to four microchromosomes each. BOE12 and BOE 14 hybridized to two microchromosomes each. BOE13 hybridized to 14 microchromosomes and BOE17-20 hybridized to 16 microchromosomes (Fig 5).

## Discussion

### Chromosomal rearrangements between *Burhinus oedicnemus* and *Charadrius collaris* and their presence in Charadriiformes

The karyotype of *Charadrius collaris* (2n = 76) is described for the first time. The species of the genus *Charadrius* cytogenetically analyzed, *Charadrius alexandrinus* and *Charadrius dubius* [28], *Charadrius hiaticula* [29], *Charadrius semipalmatus* [6], *Charadrius vociferus* [29, 30], all present the same diploid numbers (2n = 76), without noteworthy differences in chromosome morphology.

**Fig 6:**
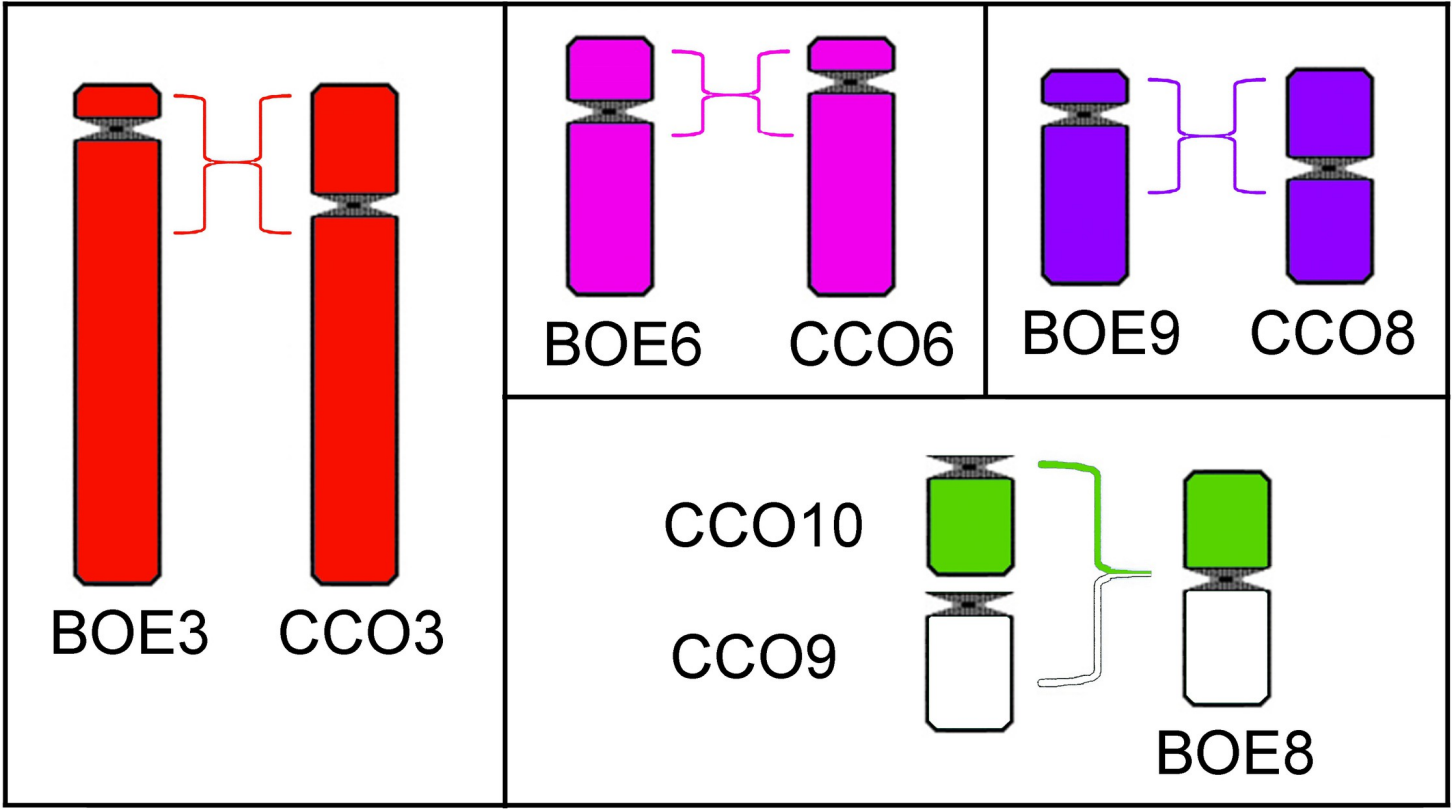
Idiogram for chromosomal pairs that have undergone pericentric inversions or centric fusions. Pericentric inversion that differ: A) BOE3 from CCO3; B) BOE6 from CCO6; C) Pericentric inversion of the metacentric CCO8 and the acrocentric BOE9; D) Fusion of CCO9 and CCO10 giving rise to BOE8.

Chromosome painting using BOE probes revealed a high degree of conservation in the first seven pairs of CCO. These data suggest that this is a characteristic common to the ancestor of the Suborder Charadrii. Some pericentric inversions and a fusion differentiate the karyotypes of CCO and BOE (Fig 6). Nie et al. [13] compared the karyotypes of BOE and GGA, and we found that the chromosomes of CCO are similar to those of GGA, so we can consider that these rearrangements occurred mostly in BOE. Many cross-hybridizations of autosomal probes also occurred on the long arm of CCOW, as already observed in *Larus argentatus* (LAR) [16]. A possible cause would be that there is a low number of interspersed repetitive sequences in autosomes and a high number in the W chromosome. Sequencing of this regions is necessary to confirm this possibility.

The homology of BOE13 with 20 micros in CCO suggests that BOE13 was formed through fusion of microchromosomes. This probe also bears the nucleolus organizing region [13] showing its presence in 1 to 4 pairs of microchromosomes, with other cross-hybridization occurring in some of them [16]. However, these results should be treated with caution since we do not use Cot1 to suppress the cross-hybridization of shared repeats present in these paints. The smallest pairs of BOE macrochromosomes appear to result from fusion of 1 to 2 pairs of microchromosomes derived from the common ancestor of Charadrii. The same can be assumed for VCH (see section below).

### Chromosomal rearrangements between *Burhinus oedicnemus* (BOE) and *Vanellus chilensis* (VCH) and their presence in Charadriiformes

The karyotype of *Vanellus chilensis* (2n = 78) has been described by classical and molecular cytogenetics [11], using GGA and *Leucopternis albicollis* Latham, 1790 (LAL) probes. In the present study we provide more thorough detail on the genome organization using BOE probes.

*Vanellus chilensis* has a karyotype typical of ancestral birds, conserved for about 100 million years, with some small variations [5]. VCH pairs 1–3, 5 and 6 remain conserved. When these results are compared with those obtained with the GGA probes for VCH, the fusion between GGA7 and GGA8 in VCH4 is observed, as reported by others [11].

Previous studies [11] show that VCH has 10 pairs of macrochromosomes and the sex chromosome Z is acrocentric, while in the present work there are only 8 pairs of macrochromosomes and a submetacentric Z. This variation in the number of macros can be explained by different opinions in the classification of macros and micros, as there is little difference in size between pair 8 (smallest pair of macrochromosomes) and pair 9 (largest pair of microchromosomes) (Fig 4). The Z chromosome issue however deserves special attention. The morphology of this chromosome in the Fig 1 shown by those authors is clearly different from that presented here. Unfortunately, they did not hybridize with a Z probe, which may raise doubts as to whether the chromosome indicated as Z really is this sex chromosome. However, if it is, it would indicate the occurrence of a pericentric inversion. There is a possibility that we are observing a population difference but, again, it is difficult to make any statement as our sample was collected in the field and those authors’ sample was a captive one, whose geographic origin is not described. In any case, the comparison of the morphology of the Z chromosome described here in VCH is different from that of CCO suggesting that inversions in the Z chromosome are not rare, as already suggested for other birds [20].

BOE13 hybridized to 14 microchromosomes and is also the bearer of a Nucleolus Organizing Region, so a variation in the number of hybridizations on VCH microchromosomes is expected [13]. BOE17-20 hybridized to 16 microchromosomes, corroborating the data obtained between BOE and GGA [13].

The 4th chromosome pair of VCH (and their homologues BOE5 and CCO5) is the result of the fusion of chromosomes GGA7 and 8 (Table 1). Therefore, as previously proposed [11], our results also support that this is an exclusive feature of Charadrii, for this fusion is not found in the other suborders, Lari (*Larus argentatus*) [16] or Scolapacii (*Jacana jacana*, [17]; *Actitis macularius*, [18]; Table 1).

**Table 1:** Chromosomal correspondence between *Gallus gallus* (GGA), *Burhinus oedicnemus* (BOE), *Vanellus chilensis* (VCH), *Charadrius collaris* (CCO), *Larus argentatus* (LAR), *Actitis macularius* (AMA), and *Jacana jacana* (JJA) demonstrated by chromosome painting. The numbers of chromosome pairs are those of the species karyotype. They are compared with the Putative Ancestral Avian Karyotype (PAK). Micro = microchromosome.? = Hybridization did not work.

PAK [4]	GGA [20]	BOE [13]	VCH (present study)	CCO (present study)	LAR [16]	AMA [18]	JJA [17]
1	1	1	1	1	1	1, 2, Wq	1
2	2	2	2	2	2	3, 11, 12, 13, Wq	4, 5p, 6p, 9
3	3	3	3	3	3	4, 14, 15, Wq	2q, 3p, 7q
4	4q	4	5	4	5	6, 16, W	2p, 3q
7, 8	7, 8	5	4	5	7, 8	7, 8	7p,6q
5	5	6	6	6	4	9, 10, Wq	5q, 8q
9	9	7 (9, R3 & R6)	6 micros, Wq	7	6, 7, 11	5, 2 micros, Wq	10
10	4p	8 (4p, R2)	8, 2 micros	9, 10	9	?	15
6	6	9 (6, 1 micro)	7	8	6, 18	8 micros, Zq, Wq	13, 14
-	-	10 (R1 & R4)	4 micros	2 micros	4, 8	17,20	-
-	-	11 (R2 & R7)	4 micros	4 micros	10, 16	18, 2 micros	-
-	-	12 (R5)	2 micros	4 micros, Wq	12, 17	19	20
-	-	13 (R6 & R9)	14 micros	20 micros, Wq	15, 25	6 micros, Wq	-
-	-	14 (R5)	2 micros	2 micros, Wq	13	2 micros, Wq	21
-	-	15, 16	6 micros, Wq	2 micros, Wq	14, 19, 23	6 micros, Wq	-
-	-	17, 18, 19, 20 (R9)	16 micros	12 micros, Wq	22, 24, 26	6 micros, Wq	-
Z	Z	Z	Z	Z	Z, Wq	Z, Wq	Z
W	W	W	W	W	Zq, Wq	W, Zq	W

Because *B*. *oedicnemus* has atypical karyotype with only four pairs of microchromosomes—most microchromosomes commonly seen in other birds had undergone fusions becoming parts of larger chromosomes. Thus, the whole chromosome probes can be useful to track the evolution of microchromosomes in other species. At the first glimpse microchromosomes may appear to be stable, but detailed analysis of hybridization signals on microchromosomes of CCO and VCH shows a considerable degree of variability. BOE10 hybridizes to 2 microchromosomes in CCO and 4 in VCH; BOE12 hybridizes with 4 microchromosomes in CCO and 2 in VCH; BOE13 hybridizes to 20 microchromosomes in CCO and 14 in VCH; BOE17-20 hybridize to 12 microchromosomes in CCO and 16 in VCH; BOE7 hybridizes with 1 pair of macrochromosome in CCO and 7 microchromosomes in VCH; BOE8 hybridizes with 1 pair of macrochromosome in CCO and with 1 pair of macrochromosome and 2 microchromosomes in VCH. Although it is not possible here to identify which pairs of microchromosomes are hybridized with each BOE probe, the variation in the number of these microchromosomes indicates a considerable number of rearrangements involving microcchromosomes coud have happened, more than that involving macrochromosomes. Studies with BAC-FISH using specific probes for microchromosomes will allow a better understanding of these rearrangements.

### Comparative analysis between the three suborders of Charadriiformes and with the order Gruiformes

**Fig 7:**
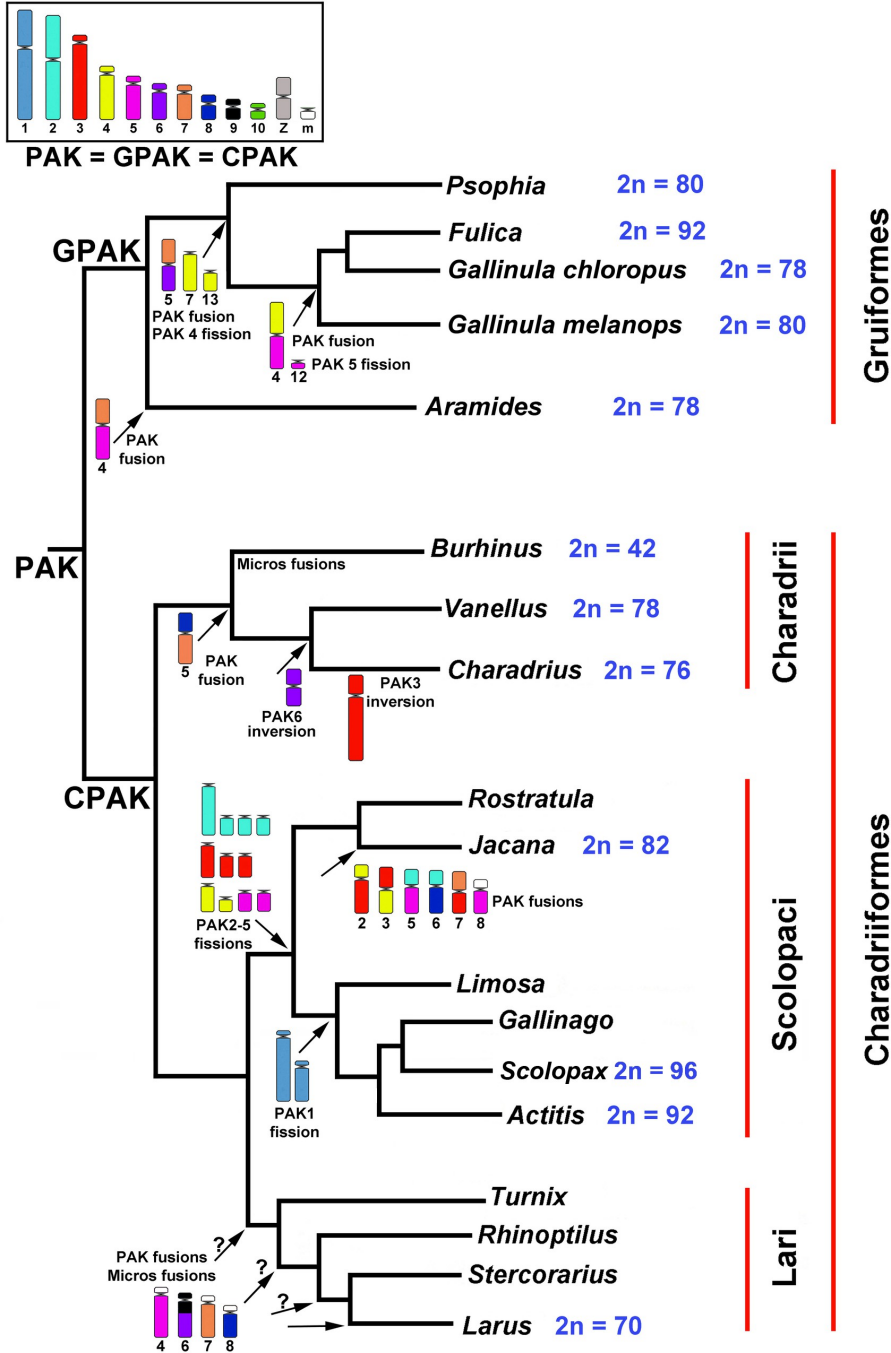
Phylogeny of Charadriiform and Gruiform Orders modified from original publications [1, 21]. Chromosomal data: Gruiformes [16, 20, 21, 31]; *Burhinus* [13]; *Vanellus* [11; present work]; *Charadrius* (present work); *Jacana* [17]; *Actitis* [18]; *Scolopax* [19]; *Larus* [16]; PAK [4]. The numbers under the rearranged chromosomes refer to the karyotypes described by the respective authors. Only the diploid numbers of the genera analyzed by molecular cytogenetics are presented. M = microchromosome;? = Doubt about the location of the rearrangements; PAK: Avian Putative Ancestral Karyotype; GPAK: Gruiformes Putative Ancestral Karyotype; CPAK: Charadriiformes Putative Ancestral Karyotype. The karyotypes CPAK and GPAK are the same as PAK because there are no common rearrangements among the analyzed clades (Gruiformes, Charadrii, Scolopaci and Lari).

Since chromosomal painting data for the three Charadriiformes suborders is already available in the literature, the data obtained here were compared to those previously described for *Gallus gallus* [22], for the avian Putative Ancestral Karyotype [4] and the Charadriiformes *Larus argentatus* [16], *Actitis macularius* [18], and *Jacana jacana* [17]. The comparisons are presented in Table 1, which shows the correspondence of homologies with BOE of those species studied only with GGA probes. We performed a comparative analysis of the rearrangements found in each suborder, using the molecular phylogeny as a guide [1], illustrated in Fig 7. As previously observed [17], we also confirm that each suborder has its own chromosomal signatures and here we detail these signatures: Charadrii (fusion PAK7 with PAK8), Scolopaci (fission of PAK 2 to 5, while a branch of this suborder has the fission of PAK1 as signature, at least with data available now [18, 19] and Lari (fusion PAK6 with PAK9, fusion of PAK7 and PAK8 with microchromosomes; pericentric inversion in PAK5). It is important to note that the supposed signatures for Lari were observed only in LAR [16], which is not a basal species in the suborder. Studies on more Lari species are needed to confirm whether these rearrangements are Lari signatures or LAR autapomorphies. Another interesting rearrangement is the fusion of PAK10 with R2 (a fusion of two microchromosomes, [13]) observed in BOE8 and LAR9, and split in CCO9 and CCO10. Since in PAK the segments are separated, their presence in Charadrii and Lari, but absence in Scolopaci, can be explained in two alternative ways: 1) The fusion happened twice, once in Charadrii and once in Lari and, therefore, Scolopaci maintains the ancestral form; 2) the fusion is a signature of Charadriiformes and should be present in the three suborders; its absence in Scolopaci could be due to a fission that separated the segments again. Since the question of fusing PAK10 with a microchromosome remains open, we have not found any rearrangements shared between the three suborders. Thus, the ancestral karyotype of Charadriiformes (CPAK, Fig 7) remains the same as the PAK, as previously suggested [17].

Since the order Gruiformes is a sister group of Charadriiformes [1], we compared our data with chromosomal painting data published for Gruiformes [16, 20, 21, 31], described for *Fulica atra* Linnaeus, 1758 (FAT), *Gallinula chloropus* Linnaeus, 1758 (GCH) [16, 20], and *Gallinula melanops* Vieillot, 1819 (GME) [31], as well as *Aramides cajaneus* Statius Muller, 1776 (ACA) and *Psophia viridis* Spix, 1825 (PVI) [21]. The determination of homologies between data obtained with GGA [20, 21] and BOE [16] chromosome probes is found in the S1 Table. Charadriiformes and Gruiformes (Fig 7) do not share any rearrangement that could be a signature of the branch from which the orders originated. Thus, the ancestral karyotype of Gruiformes (GPAK, Fig 7) could be the same as PAK and CPAK [21].

## Conclusion

We used a published molecular phylogeny [1] as a guide for studying the karyotype evolution of Charadriiformes, determining the place where each rearrangement occurred and whether each suborder shares the same chromosome signature. The suborder Charadrii has large, conserved chromosome pairs in relation to PAK. The fusion of PAK7 with PAK8 gives rise to a metacentric chromosome, which is characteristic of the Charadrii species already analyzed (BOE5; CCO5; VCH4). The comparative analysis between the Charadriiformes suborders so far does not show any shared rearrangement, indicating that each suborder followed an independent evolutionary path showing no differences in relation to the PAK. Likewise, although the orders Charadriiformes and Gruiformes are sister branches, they do not share any chromosomal rearrangement that can be considered a chromosomal signature. Chromosome painting data from more Charadriiformes species will test these propositions and give more details on the karyotypic evolution of this order.

## Supporting information

S1 Table Chromosomal correspondence between *Gallus gallus* (GGA), *Burhinus oedicnemus* (BOE), *Fulica atra* (FAT), *Gallinula chloropus* (GCH), *Aramides cajaneus* (ACA) and *Psophia viridis* (PVI) demonstrated by chromosome painting. The numbers of chromosome pairs are of the karyotype of each species. They were also compared with the Putative Ancestral Avian Karyotype (PAK). Micro = microchromosome.
(DOCX)

S1 Fig Metaphases of *Charadrius collaris* showing the hybridization of all whole chromosome probes from *Burhinus oedicnemus*. (JPG)

S2 Fig Metaphases of *Vanellus chilensis* showing the hybridization of all whole chromosome probes from *Burhinus oedicnemus*. (JPG)
