# Supplementary material for: Chromosomal painting in Charadrius collaris Vieillot, 1818 and Vanellus chilensis Molina, 1782 and an analysis of chromosomal signatures in Charadriiformes
Source: PLoS One. 2022 Aug 10;17(8):e0272836. doi: 10.1371/journal.pone.0272836 (PMC9365183; doi:10.1371/journal.pone.0272836)
Supplement: S1 Table — The numbers of chromosome pairs are of the karyotype of each species. They were also compared with the Putative Ancestral Avian Karyotype (PAK). Micro = microchromosome. (DOCX) [file pone.0272836.s001.docx]

Table S1: Chromosomal correspondence between *Gallus gallus* (GGA)*, Burhinus oedicnemus* (BOE), *Fulica atra* (FAT), *Gallinula chloropus* (GCH), *Gallinula melanops* (GME), *Aramides cajaneus* (ACA) and *Psophia viridis* (PVI) demonstrated by chromosome painting. The numbers of chromosome pairs are the ones of the karyotype of each species. They were also compared with the Putative Ancestral Avian Karyotype (PAK). micro = microchromosome.

| **PAK**  **[4]** | **GGA**  **[20]** | **BOE**  **[13]** | **FAT**  **[16]** | **GCH**  **[20]** | **GME [31]** | **ACA**  **[21]** | **PVI**  **[21]** |
| --- | --- | --- | --- | --- | --- | --- | --- |
| 1 | 1 | 1 | 1 | 1 | 1 | 1 | 1 |
| 2 | 2 | 2 | 2 | 2 | 2 | 2 | 2 |
| 3 | 3 | 3 | 3 | 3 | 3 | 3 | 3 |
| 4 | 4q | 4 | 4p, 6 | 4p, 6 | 4p, 6 | 5 | 5 |
| 7, 8 | 7, 8 | 5 | 5p, 7, 33 | 5p, 6 | 5p, 6 | 4p, 8 | 4p, 7 |
| 5 | 5 | 6 | 4q, 10 | 4q, 12 | 4q, 12 | 4q | 6 |
| 9 | 9 | 7p | 9 | 8 | 8 | 9 | 8 |
| 10 | 4p | 8p | 8 | 7, 13 | 7, 13 | 7 | 9, 11 |
| 6 | 6 | 9q | 5q | 5q | 5q | 6 | 4p |
| - | - | 10 | 12, 16 | - | - | - | - |
| - | - | 11 | 11, 22 | - | - | - | - |
| - | - | 12 | 15, 20 | - | - | - | - |
| - | - | 13 | 17, 21, 31 | - | - | - | - |
| - | - | 14 | 18 | - | - | - | - |
| - | - | 15, 16 | 19, 26, 34 | - | - | - | - |
| - | - | 17, 18, 19, 20 | 23, 28, 32 | - | - | - | - |
|  | R1 (10, 11, 12)* | 10q | 12 or 16 |  |  |  |  |
|  | R2 (10, 11, 12)* | 8q, 11q | 14, 11 or 22 |  |  |  |  |
|  | R3 (13)* | 7q | 13 or 25 |  |  |  |  |
|  | R4 (13, 14, 15 20)* | 10p | 12 or 16 |  |  |  |  |
|  | R5 (10, 11, 12)* | 12q, 14p | 15 or 20; 18 |  |  |  |  |
|  | R6 (17, 18, 19)* | 7q, 13q | 13 or 25; 17, 21 or 31 |  |  |  |  |
|  | R7 (25, 27)* | 11p | 11 or 22 |  |  |  |  |
|  | R9 (28)* | 13p, 17, 18, 19, 20 | 17, 21 or 31; 23, 28 or 32 |  |  |  |  |
| Z | Z | Z | Z, Wq | Z | Z | Z | Z |
| W | W | W | Z, Wq | W | W | W | W |

*The Rs are pools of microchromosomes [13] and the numbers in parentheses indicate which GGA microchromosomes are found in each R [29].
