# Supplementary figures and images for: Chromosomal painting in Charadrius collaris Vieillot, 1818 and Vanellus chilensis Molina, 1782 and an analysis of chromosomal signatures in Charadriiformes
Source: PLoS One. 2022 Aug 10;17(8):e0272836. doi: 10.1371/journal.pone.0272836 (PMC9365183; doi:10.1371/journal.pone.0272836)

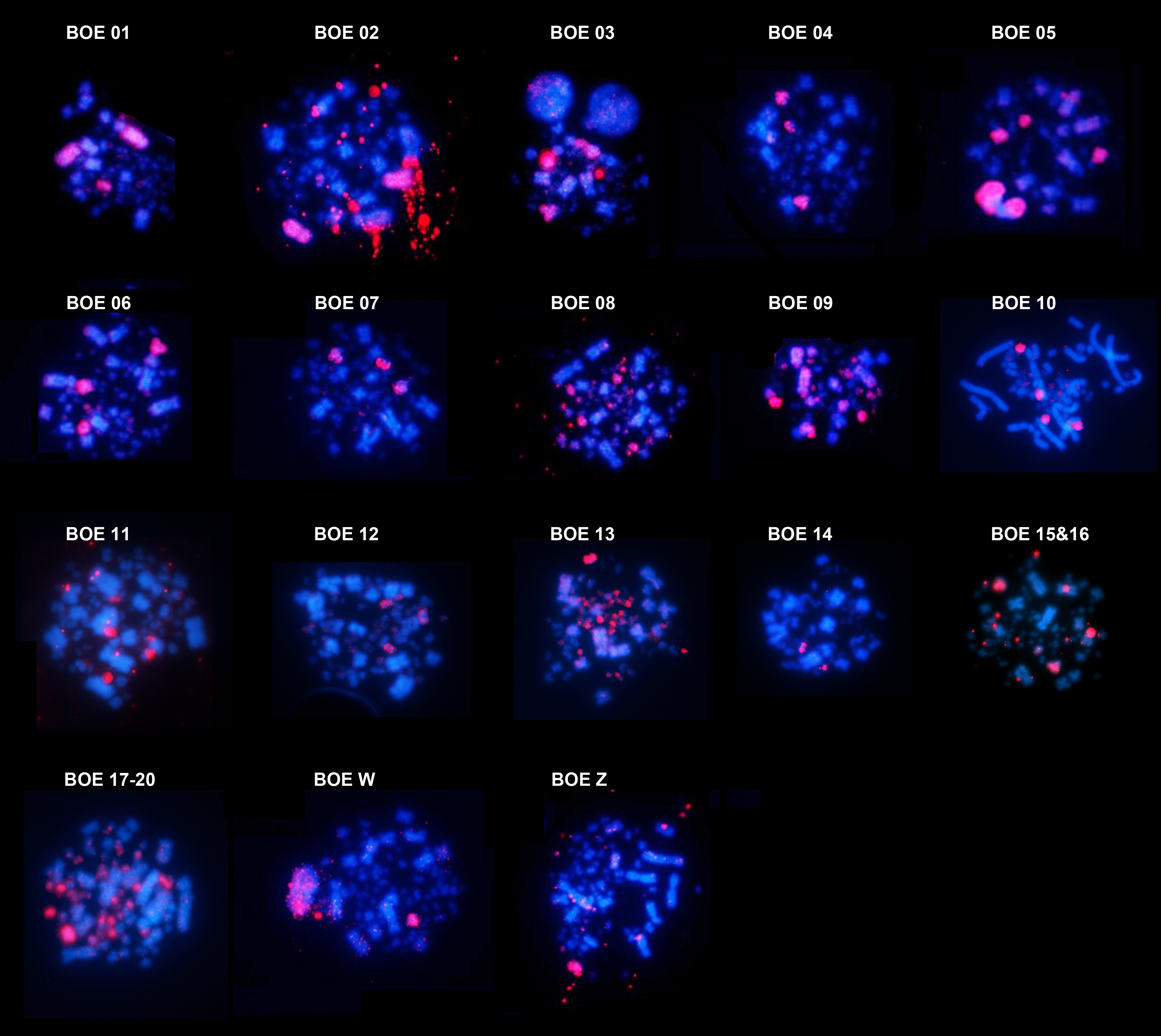

Supplement: S1 Fig — (JPG) [file pone.0272836.s002.jpg]

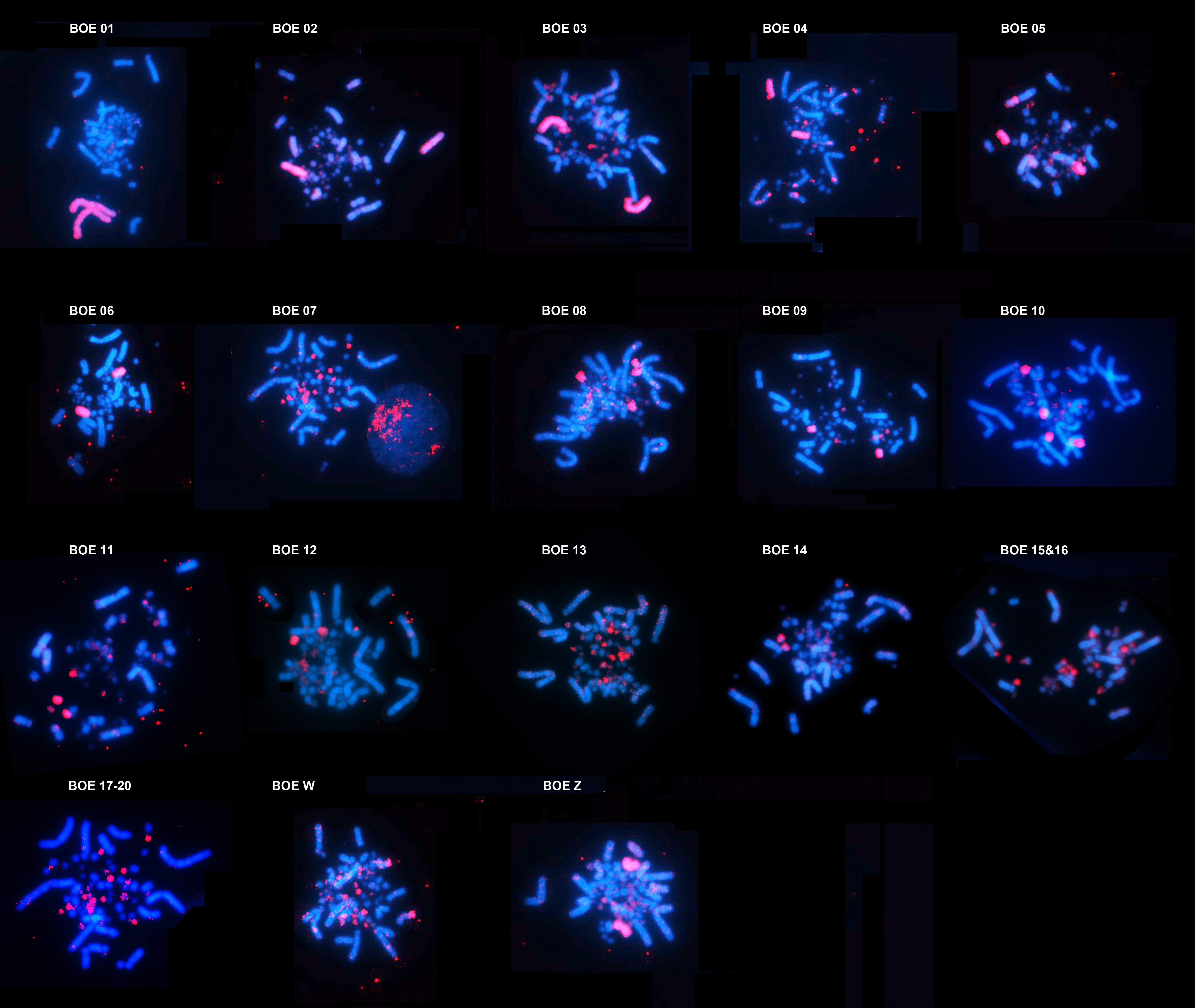

Supplement: S2 Fig — (JPG) [file pone.0272836.s003.jpg]
